# The miR‐223/nuclear factor I‐A axis regulates inflammation and cellular functions in intestinal tissues with necrotizing enterocolitis

**DOI:** 10.1002/2211-5463.13164

**Published:** 2021-06-01

**Authors:** Yu Zheng Wu, Kathy Yuen Yee Chan, Kam Tong Leung, Hugh Simon Lam, Yuk Him Tam, Kim Hung Lee, Karen Li, Pak Cheung Ng

**Affiliations:** ^1^ Department of Paediatrics Prince of Wales Hospital The Chinese University of Hong Kong Hong Kong; ^2^ Department of Surgery Prince of Wales Hospital The Chinese University of Hong Kong Shatin Hong Kong

**Keywords:** apoptosis, cell proliferation, miR‐223, necrotizing enterocolitis, *NFIA*

## Abstract

We previously demonstrated that microRNA(miR)‐223 is overexpressed in intestinal tissue of infants with necrotizing enterocolitis (NEC). The objective of the current study was to identify the target gene of miR‐223 and to investigate the role of the miR‐223/nuclear factor I‐A (*NFIA*) axis in cellular functions that underpin the pathophysiology of NEC. The target gene of miR‐223 was identified by *in silico* target prediction bioinformatics, luciferase assay, and western blotting. We investigated downstream signals of miR‐223 and cellular functions by overexpressing the miRNA in Caco‐2 and FHs74 cells stimulated with lipopolysaccharide or lipoteichoic acid (LTA). *NFIA* was identified as a target gene of miR‐223. Overexpression of miR‐223 significantly induced *MYOM1* and inhibited *NFIA* and *RGN* in Caco‐2 cells, while costimulation with LTA decreased expression of *GNA11*, *MYLK,* and *PRKCZ*. Expression levels of *GNA11, MYLK, IL‐6,* and *IL‐8* were increased, and levels of *NFIA* and *RGN* were decreased in FHs74 cells. These potential downstream genes were significantly correlated with levels of miR‐223 or *NFIA* in primary NEC tissues. Overexpression of miR‐223 significantly increased apoptosis of Caco‐2 and FHs74 cells, while proliferation of FHs74 was inhibited. These results suggest that upon binding with *NFIA*, miR‐223 regulates functional effectors in pathways of apoptosis, cell proliferation, G protein signaling, inflammation, and smooth muscle contraction. The miR‐223/*NFIA* axis may play an important role in the pathophysiology of NEC by enhancing inflammation and tissue damage.

## Graphical abstract

MicroRNA (miR)‐223 is overexpressed in the bowel of infants with necrotizing enterocolitis (NEC). This study investigated the role of miR‐223 in cellular functions that underpin the pathophysiology of NEC. Through identifying direct and downstream targets, and using a gain‐of‐function approach, we demonstrated that the miR‐223/NFIA axis could regulate NEC pathophysiology by interacting with multiple pathways that aggravate inflammation and tissue damage.

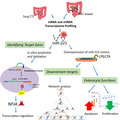

**Abbreviations**
GAPDH: Glyceraldehyde 3‐phosphate dehydrogenase
GLuc: Gaussia luciferase
GNA11: G protein subunit alpha 11
ICAM1: Intercellular adhesion molecule 1
IL‐6: Interleukin‐6
IL‐8: Interleukin‐8
LPS: Lipopolysaccharides
LTA: Lipoteichoic acid
MYLK: Myosin light chain kinase
MYOM1: Myomesin 1
NEC: Necrotizing enterocolitis
NFIA: Nuclear factor I‐A
PRKCZ: Protein kinase C zeta
RGN: Regucalcin
TLR2: Toll‐like receptor‐2
TLR4: Toll‐like receptor‐4
TNF: Tumor necrosis factor

Necrotizing enterocolitis (NEC) is a devastating gastrointestinal (GI) disease that is strongly associated with prematurity. It is one of the leading causes of neonatal morbidity and mortality among very low birthweight infants (< 1500 g), with an incidence of 6–10% [1, 2] and a mortality of 20–30%. Infants who survive are at risk of long‐term complications, including short bowel syndrome, parenteral nutrition‐associated cholestasis, poor physical growth, and neurodevelopmental impairment [3, 4]. Despite decades of NEC research, its etiology and pathophysiology remain incompletely understood. Multiple mechanisms and contributing factors have been proposed, including immature gut mucosa with impaired host defense immunity, enteral feeding with abnormal bacterial colonization, and other conditions that can result in a bacterial/inflammatory response that can lead to subsequent tissue damage [5, 6]. In our recent series of studies on expressional arrays of NEC tissues, we identified aberrant mRNA expression patterns, which suggested a complex mechanism that was initiated at receptor *TLR4*, mediated *via* key transcription factors *NFKB2*, AP‐1*/FOSL1*, *FOXA1*, and *HIF1A*, and extended to downstream pathways involving angiogenesis, arginine metabolism, cell adhesion and chemotaxis, extracellular matrix remodeling, hypoxia/oxidative stress, inflammation, and muscular functions [7]. We also reported the first differential microRNA (miRNA) expression array of NEC tissues and provided evidence that miR‐431 and its target gene *FOXA1* could play a key role in intensifying the inflammatory responses in NEC tissues [8, 9]. In a systematic large‐scale meta‐analysis study, the miRNA‐429/200a/b and miRNA‐141/200c clusters and their target genes have been suggested as potential biomarkers for NEC [10]. In addition, we reported elevated plasma levels of miR1290 in infants with NEC and suggested that miR1290 could be developed as a biomarker for early diagnosis of NEC [11].

miRNAs are a class of small, single‐stranded noncoding RNA molecules (18–24 nucleotides) that can suppress translation and stability of transcripts through partial complementarity to the 3′‐UTRs of protein‐coding mRNAs [12]. Emerging evidence has revealed that miRNAs play important functions in normal cellular and physiologic events, and their dysregulation could be associated with diverse pathologic conditions, including cancer [13], chronic infection, inflammation [14], and acute organ injury [15]. In our previous study, we reported that miR‐223 expression was upregulated 25.16‐fold in intestinal tissues of infants with NEC compared with noninflammatory surgical control (Surg‐CTL) tissues [8]. miR‐223 has been reported to play vital roles in the development and progression of inflammatory conditions [16, 17, 18, 19]. We propose that the marked upregulation of miR‐223 in NEC could be associated with a disturbance of intestinal cell homeostasis and NEC pathophysiology. Our objectives were to identify the target gene of miR‐223 in NEC tissues and to investigate the role of the miR‐223/nuclear factor I‐A (*NFIA*) axis in intestinal cell models upon exposure to bacterial toxins.

## Results

### Clinical characteristics of recruited infants

As previously reported, there were no statistically significant differences in demographic and clinical characteristics, for example, sex, gestational age, and birthweight, between the infants recruited to the NEC and Surg‐CTL groups (Table S1). However, as expected, infants with NEC had significantly longer duration of hospitalization (*P* = 0.014) [8].

### Increased expression of miR‐223 in NEC tissues and identification of target gene

**Fig. 1:**
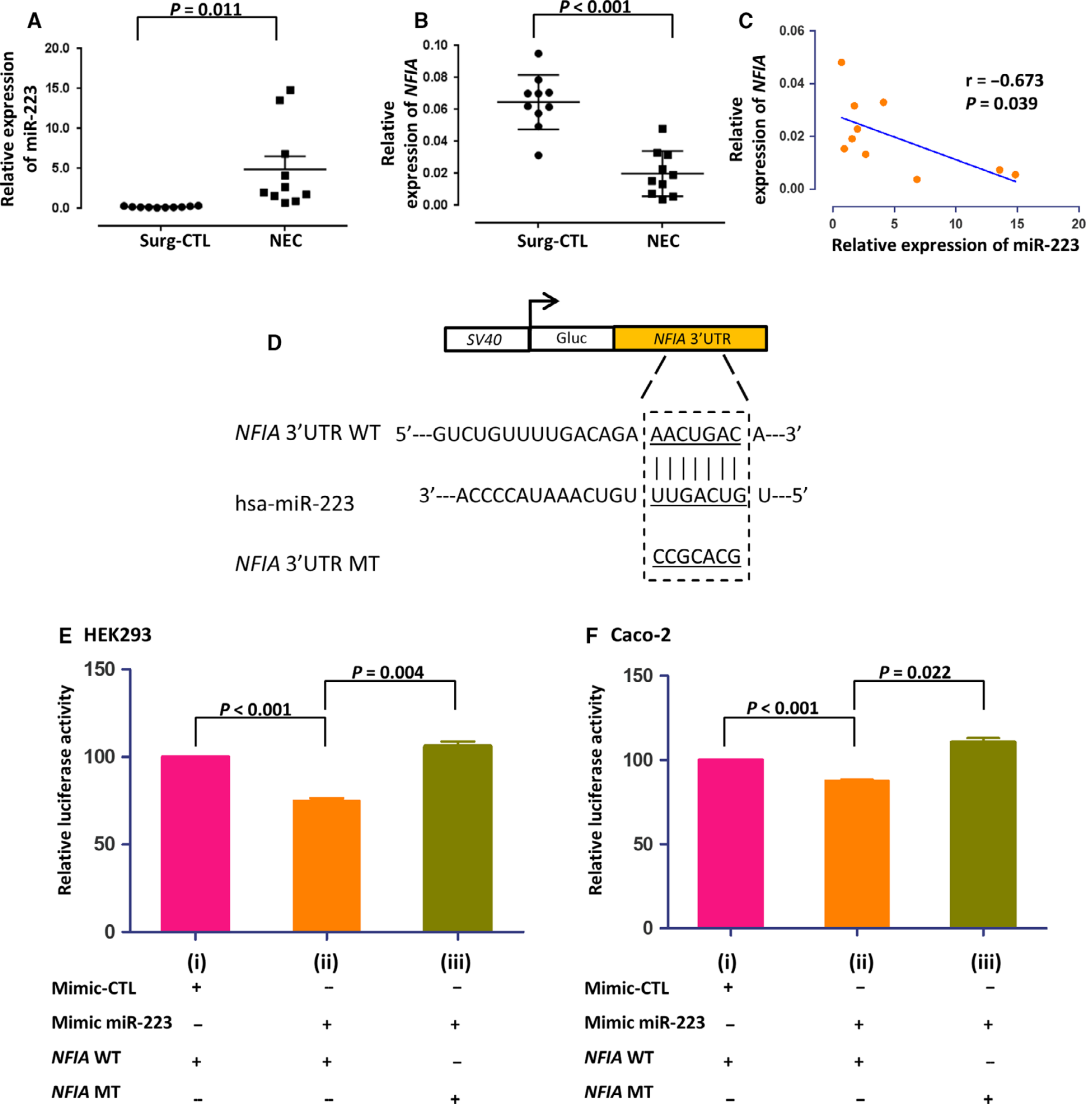
Expression of miR‐223 and *NFIA* in NEC tissues and direct binding of miR‐223 and *NFIA* by luciferase reporter assay.(A) Relative expression level of miR‐223 to U6 and (B) relative expression of *NFIA* to GAPDH in human Surg‐CTL and NEC tissues were quantified by qPCR (*n* = 10 for each group). (C) Correlation between miR‐223 and *NFIA* in NEC tissues. Correlation analysis was performed by the Spearman correlation test. *r* represents the Spearman correlation coefficient. *n* = 10 for each group. (D) Seeding site of hsa‐miR‐223 and mutation sequence of the *NFIA* 3’UTR construct. (E) HEK293 cells were cotransfected with (i) mimic‐CTL or (ii) mimic miR‐223 and wild‐type *NFIA* 3’UTR (WT) or (iii) mimic miR‐431 with mutated type NFIA 3′UTR (MT). Luciferase activity was measured at 48 h after transfection. (F) The same experimental procedure was performed on Caco‐2 cells. Statistical analysis was performed using paired *t‐*test, two‐sided. Data were presented as mean ± SEM, *n* = 4.

Expression of miR‐223 was upregulated in small intestinal tissues of NEC (*n* = 10) compared with Surg‐CTL infants (*n* = 10 in each group; 25.16‐fold, *P* < 0.001; Fig. 1A) [8]. *In silico* target prediction revealed that *NFIA* was among the top candidate target genes of miR‐223 (Table S2). Furthermore, *NFIA* was decreased by 69.15% in NEC tissues compared with Surg‐CTL tissues (*P* < 0.001; Fig. 1B). In NEC tissues, there was an inverse correlation between mRNA levels of miR‐223 and *NFIA* (Fig. 1C).

The direct binding of miR‐223 and 3’UTR of *NFIA* was validated in HEK293 cells by luciferase assay in the presence of mimic miR‐223 or mimic‐CTL. The luciferase activity of HEK293 cells harboring the *NFIA* wild‐type (WT) 3’UTR reporter plasmid (Fig. 1D) was inhibited by 25.1% in the presence of mimic miR‐223 [*n* = 4; *P* < 0.001; Fig. 1E(ii)], compared with that containing the mimic‐CTL [Fig. 1E(i)]. Replacing *NFIA* WT 3’UTR sequence with mutated nucleotides (MT) at the seeding region of miR‐223 [Fig. 1E(iii)] or the presence of the irrelevant miR‐431 (Fig. S1) did not alter the luciferase activity. Direct binding between miR‐223 and 3’UTR of *NFIA* was further confirmed in the human colorectal cell line Caco‐2. The luciferase activity was decreased by 12.4% in the presence of mimic miR‐223 and WT *NFIA* plasmid [*n* = 4, *P* < 0.001; Fig. 1F(ii)], but not in the presence of binding site‐mutated plasmid (Fig. 1F(iii)).

### Overexpression of miR‐223 decreased NFIA expression in Caco‐2 cells

**Fig. 2:**
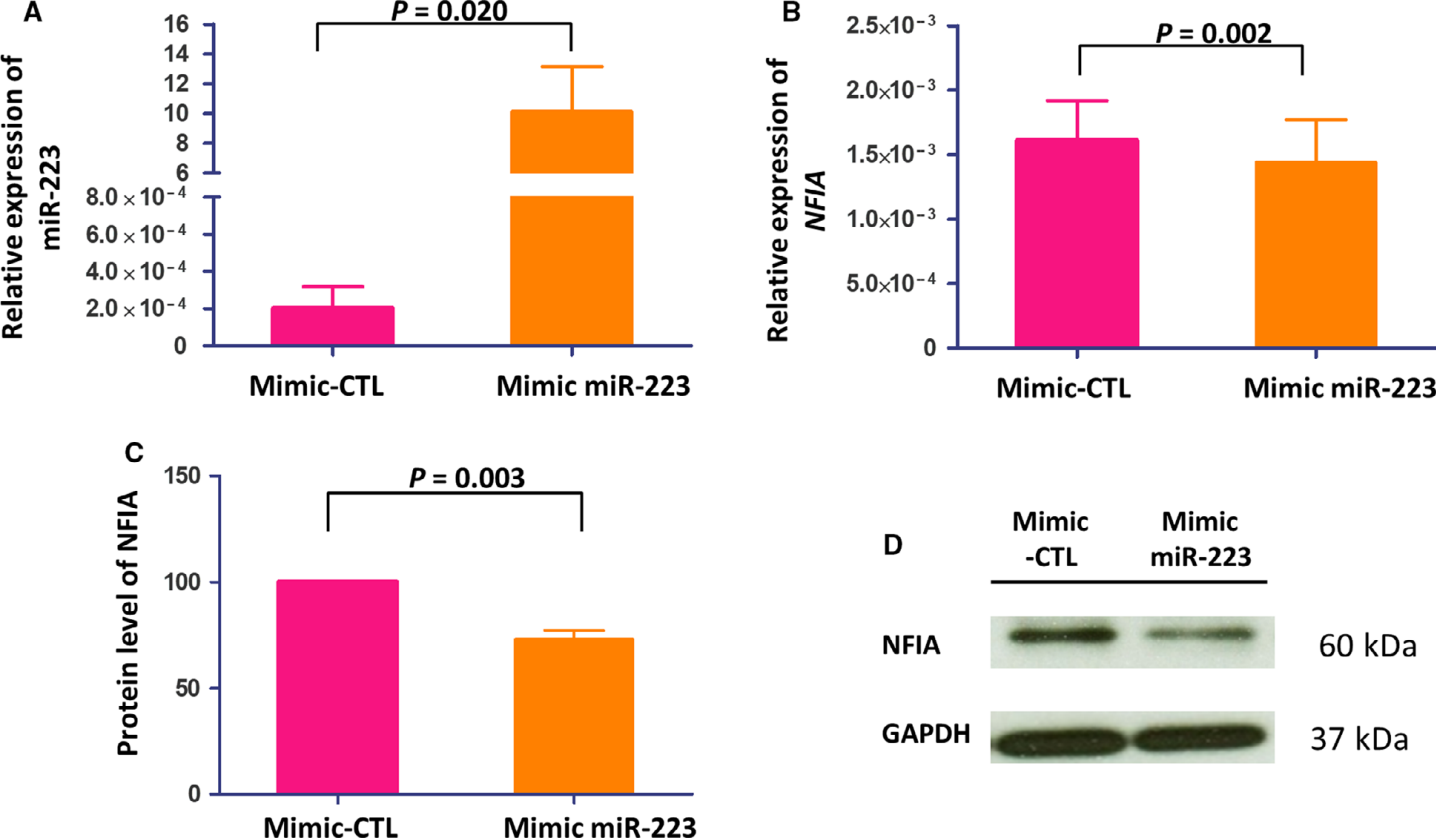
Relative expression level of miR‐223 and *NFIA* in Caco‐2 cells upon overexpression of mimic miR‐223. (A) Relative expression of miR‐223 to U6 and (B) *NFIA* to GAPDH in Caco‐2 cells after transfection with mimic‐CTL and mimic miR‐223 by qPCR (*n* = 6). Expressions of miR‐223 and *NFIA* were normalized with U6 and GAPDH, respectively. (C, D) The protein level of NFIA was measured by western blot analysis after overexpression of mimic miR‐223. Statistical analysis was performed by paired *t*‐test, two‐sided. The data were presented as mean ± SEM.

Transfection of mimic miR‐223 into Caco‐2 cells increased miR‐223 compared with cells transfected with mimic‐CTL (*n* = 6, *P* = 0.02; Fig. 2A) and suppressed *NFIA* expression by 10.6% (*n* = 6, *P* = 0.002; Fig. 2B). In accordance with mRNA expression, western blot analysis demonstrated that the NFIA protein level was decreased by 27.1% in Caco‐2 cells overexpressing miR‐223 (*n* = 5, *P* = 0.003; Fig. 2C,D).

### Correlation mRNA levels of miR‐223 and NFIA with dysregulated gene expressions in NEC tissues

**Fig. 3:**
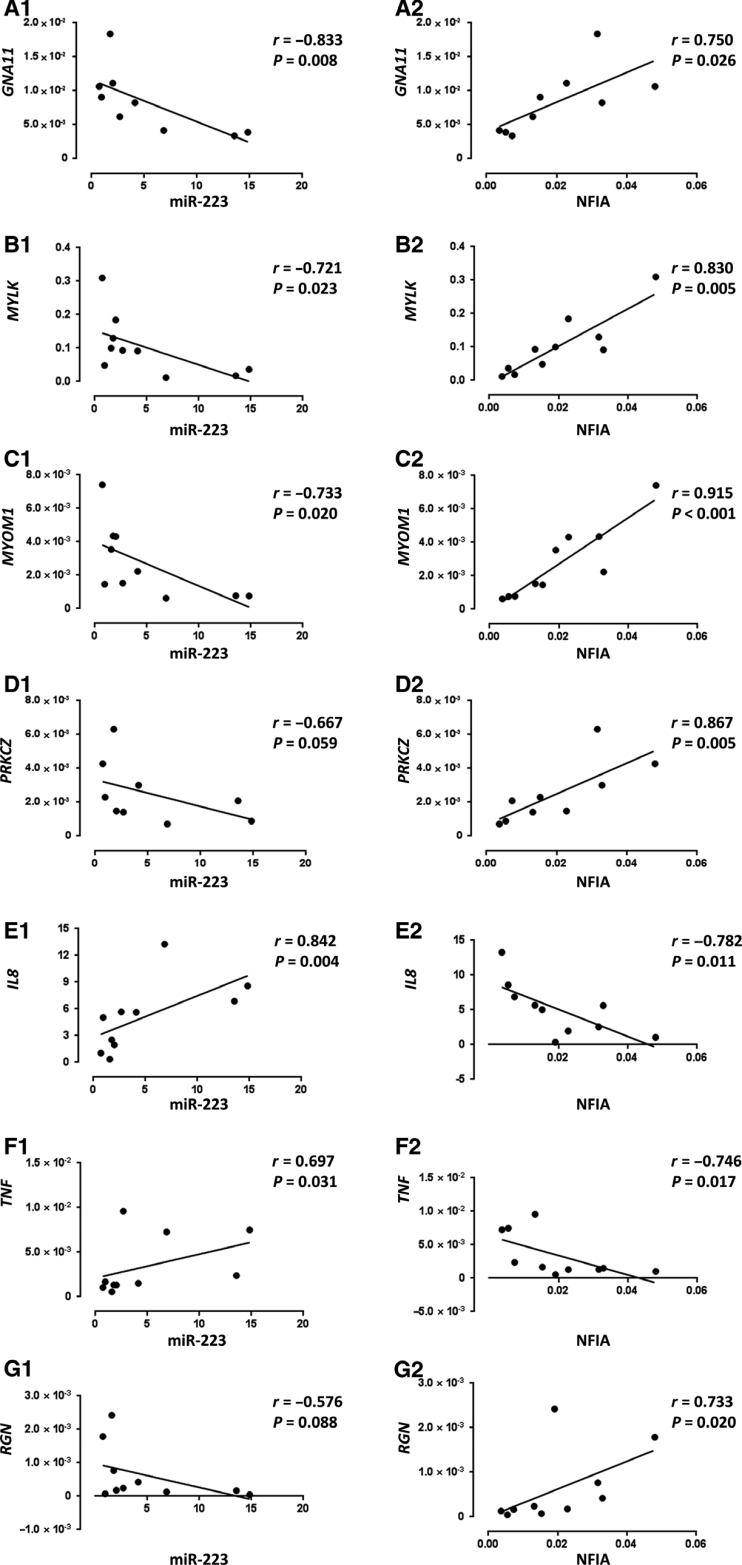
Correlation between miR‐223 or *NFIA* and miR‐223/*NFIA*‐targeted downstream genes in NEC tissues. (A1‐G1) Correlation between miR‐223 and miR‐223/*NFIA*‐targeted downstream genes in NEC tissues. (A2‐G2) Correlation between *NFIA* and miR‐223/*NFIA*‐targeted downstream genes in NEC tissues. Nonparametric correlation analysis was performed by Spearman’s correlation test. *r* represents the Spearman correlation coefficient. *n* = 10 for each group, except A1, A2 and D1, D2 (*n* = 9).

Correlation analysis was performed on levels of miR‐223 and *NFIA* with 52 dysregulated genes in intestinal tissues of infants with NEC reported in our previous study (*n* = 9–10) [7]. We observed inverse correlations between expression levels of miR‐223 and *GNA11, MYLK*, or *MYOM1* (*P* < 0.05), and positive correlations between miR‐223 and *IL8* or *TNF* (*P* < 0.05) (Fig. 3). A trend of inverse correlation was observed between miR‐223 and *PRKCZ* (*P* = 0.059) or *RGN* (*P* = 0.088). The expression levels of *NFIA* were positively correlated with *GNA11, MYLK, MYOM1*, *PRKCZ or RGN* (*P* < 0.05) and inversely correlated with *IL8* or *TNF* (*P* < 0.05). There was no significant correlation between miR‐223 and *NFIA* with *IL6* (Fig. S2).

### Effects of overexpressing miR‐223 on gene regulation in Caco‐2 and FHs74 cells

**Fig. 4:**
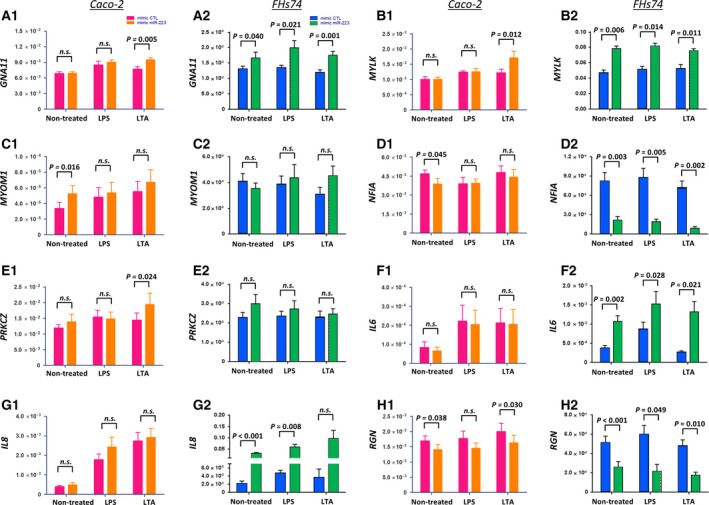
Relative expression of miR‐223/*NFIA*‐targeted downstream genes in Caco‐2 cells and FHs74 cells. (A1–H1) Caco‐2 cells were transfected with mimic‐CTL or mimic miR‐223, and stimulated with LPS or LTA (*n* = 5). (A2–H2) FHs74 cells were transfected with mimic‐CTL or mimic miR‐223, and stimulated with LPS or LTA (*n* = 5). All gene expressions were normalized with U6/ *GAPDH*. Statistical analysis was performed by paired *t*‐test, two‐sided. The data were presented as mean ± SEM.

In Caco‐2 cells, overexpression of miR‐223 increased *MYOM1* 1.5‐fold (*P* = 0.016; Fig. 4C1) and decreased *NFIA* by 16.8% (*P* = 0.045; Fig. 4D1), or *RGN* by 16.4% (*P* = 0.038; Fig. 4H1). The effects of overexpressing miR‐223 on these gene expressions appeared to be unaffected by additional exposure to Lipopolysaccharides (LPS) or lipoteichoic acid (LTA). Levels of *GNA11* (Fig. 4A1), *MYLK* (Fig. 4B1), and *PRKCZ* (Fig. 4E1) were not changed in miR‐223‐overexpressing cells but were increased upon additional exposure to LTA by 1.2‐ to 1.4‐fold (*P* < 0.05). IL‐8 was increased by exposure to LPS or LTA by 4.5‐ to 7.0‐fold, but no effect of miR‐223 was observed in all conditions (Fig. 4G1).

In FHs74 cells, overexpression of miR‐223 significantly increased *GNA11* (1.3‐fold; Fig. 4A2), *MYLK* (1.7‐fold; Fig. 4B2), *IL‐6* (2.8‐fold; Fig. 4F2), and *IL‐8* (14.1‐fold; Fig. 4G2) and decreased *NFIA* by 73.6 % (Fig. 4D2) or *RGN* by 49.2 % (Fig. 4H2, *n* = 5, *P* < 0.05). *MYOM1* (Fig. 4C2) and *PRKCZ* (Fig. 4E2) were not affected by miR‐223 overexpression. The presence of LPS or LTA also did not further alter the effects of miR‐223. Expression of *TNF* was undetectable in FHs74 cells and in Caco‐2 was not affected by overexpression of miR‐223 (Fig. S3). In intestinal tissues of NEC patients, we observed that expression levels of *GNA11, MYLK, MYOM1, NFIA, PRKCZ,* and *RGN* were significantly downregulated (*P* < 0.02), with *IL‐6* and *IL‐8* significantly increased, compared with those from Surg‐CTL subjects (*n* = 10 for each group; Fig. S4).

### Effects of overexpressing miR‐223 on proliferation and apoptosis of Caco‐2 and FHs74

**Fig. 5:**
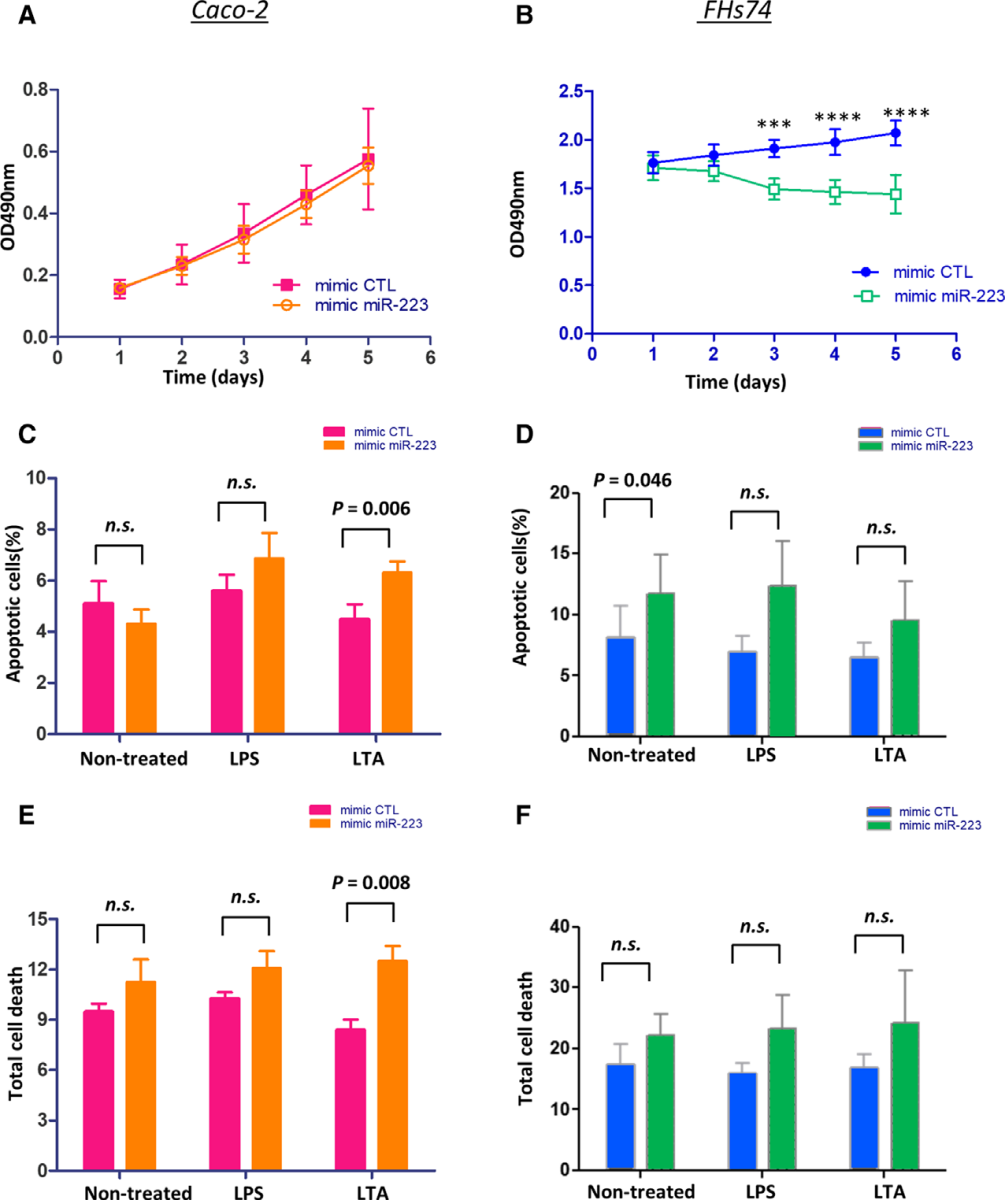
Effects of miR‐223 on cell proliferation and apoptosis of Caco‐2 cells and FHs74 cells. (A, B) Cell proliferation of Caco‐2 cells and FHs74 cells was assessed by MTS assay after transfection with mimic‐CTL or mimic miR‐223 for 5 days. Statistical analysis was performed by two‐way ANOVA with Bonferroni’s correction (*n* = 5). The data were presented as mean ± SEM. (C–F) Percentage of apoptosis and total cell death were assessed by flow cytometry upon overexpression of mimic miR‐223 and stimulation with LPS or LTA. Statistical analysis was performed by paired *t*‐test (*n* = 6), and the data were presented as mean ± SEM.***P < 0.001; ****P < 0.0001.

Overexpression of miR‐223 did not affect proliferation (Fig. 5A) or apoptotic/total cell death of Caco‐2 cells. In the additional presence of LTA, apoptosis (Fig. 5C) and total cell death (Fig. 5E) were increased 1.4‐ and 1.5‐fold (*n* = 6, *P* < 0.01; Fig. S5), respectively.

Overexpression of miR‐223 inhibited proliferation of FHs74 by 30.4 % after 5 days of culture (*n* = 5, *P* < 0.001; Fig. 5B). miR‐223 increased apoptotic cell death 1.4‐fold (*n* = 6, *P* = 0.046; Fig. 5D), and total cell death was not significantly affected (Fig. 5F). The additional presence of LPS or LTA was not associated with further effects on the cell death data.

### Proposed regulatory network of miR‐223/NFIA in NEC

**Fig. 6:**
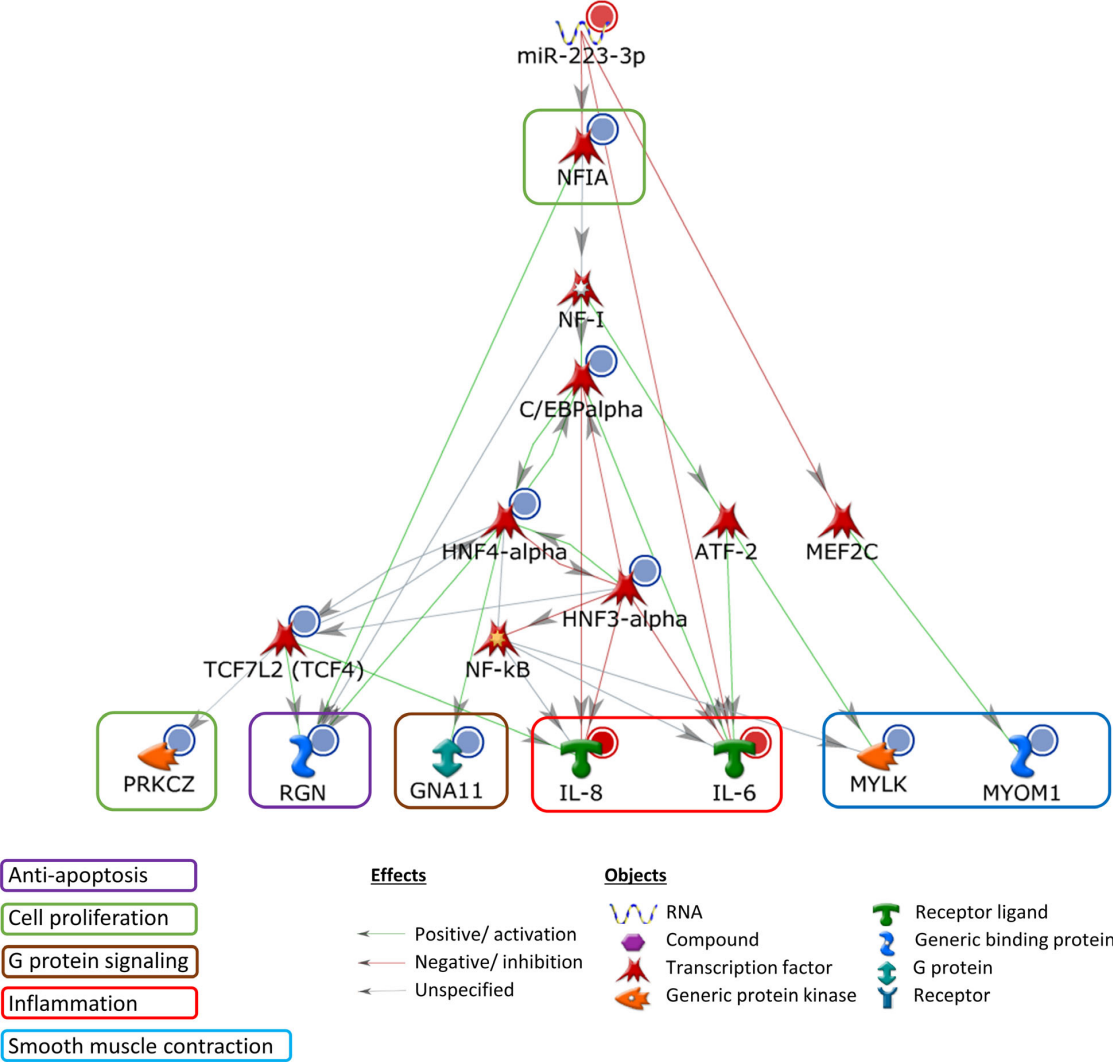
Proposed regulatory network of miR‐223/*NFIA* in NEC.A regulatory network of targeted genes downstream of the miR‐223/*NFIA* axis was generated by the MetaCore software. The red and blue circles represented upregulation and downregulation of gene expression from NEC array experiment (GEO:GSE46619). The green, red, and gray arrows represented positive, negative, and unspecified interactions between two objects. The effector genes were categorized into different functional groups, including cell apoptosis, proliferation, G protein, inflammation, and smooth muscle contraction signals, which were highlighted in different colors.

MetaCore pathway analysis suggested dysregulation of effector genes in functional categories of cell apoptosis (*RGN*), proliferation (*NFIA*, *PRKCZ*), G protein *(GNA11),* inflammation *(IL‐6, IL‐8)* and smooth muscle contraction *(MYOM1, MYLK)* signals, with possible links *via* miR‐223/NFIA and C/EBP‐α to multiple downstream transcription factors: HNF4‐α, HNF3‐α, ATF‐2, MEF2C, TCF4, and NF‐κB (Fig. 6).

## Discussion

Our study presents the first evidence that the miR‐223/*NFIA* axis is aberrantly expressed in intestinal tissues of infants with NEC and suggests that affected downstream signals could reprogram the expression of effector genes in multiple categories of cellular functions, including apoptosis, proliferation, G protein signaling, inflammation, and smooth muscle contraction. We observed increased apoptosis and decreased proliferation of intestinal cells overexpressing miR‐233 upon exposure to bacterial toxins in culture. These findings strongly suggest that dysregulation of miR‐223 and its target gene *NFIA* could contribute to the pathophysiology of NEC through escalated inflammation, increased apoptosis, and suppressed cell proliferation, which leads to irreparable tissue damage.

miR‐223 is a highly conserved miRNA known to be involved in the regulation of hematopoiesis, immune response, and inflammation [20, 21, 22]. It exerts its influence through binding to a variety of target genes in specific tissues and pathologic conditions. We report upregulation of miR‐223 in intestinal tissue of infants with NEC and identified the transcription factor *NFIA* as a binding target of miR‐223. Our observation is in line with reports on hematopoietic cells and the central nervous system in which the miR‐223/*NFIA* axis is suggested to play important roles in regulating development, cell proliferation, and malignant progression [16, 17, 23]. While not previously elaborated in NEC tissues, upregulation of miR‐223 and potential target genes involving TLR and NF‐κB signaling has been reported in the inflamed colonic mucosa of patients with ulcerative colitis (UC) [18]. SchÖnauen *et al*. demonstrated that levels of circulatory and fecal miR‐223 correlated with disease activity and could be potential biomarkers for inflammatory bowel diseases (IBD) [24]. In the mouse model of induced colitis, treatment with miR‐223 antagomir significantly improved acute intestinal conditions. The effect was suggested to be mediated by reactivating claudin‐8 and preserving the integrity of the gut epithelial barrier [22]. miR‐223 has been reported to serve both pro‐inflammatory [22, 25, 26] and anti‐inflammatory [27, 28, 29] functions, possibly dependent on specific target genes and disease conditions. In the FHs74 model, we observed upregulated expression of two pro‐inflammatory cytokines, *IL6* and *IL8*, upon overexpression of miR‐223, suggesting potent pro‐inflammatory roles of miR‐223 in NEC. Our results are consistent with those reported about CMT93 cells where overexpression of miR‐223 induced expression of inflammatory cytokines TNF‐α, IL‐6, and COX‐2, possibly mediated by FOXO3a and NF‐κB activities [25]. *NFIA* has been acknowledged as a master molecular transcriptome regulator that modulates myeloid differentiation and maturation and acts as an immune defense mechanism during sepsis [30]. Nevertheless, its implications in NEC or other inflammatory bowel conditions have not been reported.

The aberrant colonization of Gram‐positive (e.g., *Clostridium* spp.) or Gram‐negative bacteria (e.g., *E. Coli* and *Klebsiella* spp.) in the gut is an important risk factor of NEC. 30% of infants with NEC also suffer from neonatal sepsis [31]. In this context, our study has further elucidated the effects of miR‐223 overexpression on downstream gene expression signals and functions upon exposure of gut epithelial cells to LPS and LTA in culture. Our results showed upregulation of *GNA11*, *MYLK*, *MYOM1*, and *IL‐8*, and downregulation of *NFIA* and *RGN* in Caco‐2 and/or FHs74 cells upon overexpression of miR‐223. *GNA11, MYLK,* and *PRKCZ* were increased in Caco‐2 cells with the additional presence of LTA. Importantly, these affected genes are known to play important roles in the maintenance of intestinal homeostasis, including regulation of cellular differentiation and proliferation, apoptosis, smooth muscle contraction, epithelial barrier permeability, and tight junction integrity, as well as immune and inflammatory responses. A brief summary of their relevant functions is described in Table S3. Furthermore, expression levels of these regulated genes were correlated with levels of miR‐223 or *NFIA* in tissues of patients with NEC. We also observed significant dysregulation of these signals in NEC tissues compared with the Surg‐CTL counterparts. In line with the regulatory pattern of the cell models, *IL8* and *IL‐6* were upregulated in NEC tissues, while *NFIA* and *RGN* were decreased. Network analysis demonstrated that miR‐223 could downregulate target gene *NFIA* and mediate through multiple transcription factors, of which some are reported targets of miR‐223 (e.g., *FOXA1, MEF2C, HNF4‐α, C/EBP‐α*, and *NF‐κB*) [16, 32, 33]. Furthermore, the dysregulation of downstream transcription factors, FOXA1, C/EBP‐α, HNF4‐α, ATF2, HNF3‐α, TCF4, and NF‐κB, has been implicated in inflammatory GI conditions such as NEC and IBD [7, 34, 35, 36, 37, 38].

While both Caco‐2 and FHs74 have been applied as cell models for studying NEC biology [39, 40, 41], we did observe some differences in the response of these two cell lines to miR‐223 overexpression and bacterial toxin exposure, possibly due to intrinsic variations of tissue origin, disease condition, and expression level of regulatory genes and receptors. For example, we observed significantly lower or undetectable expression levels of *TNF*, Toll‐like receptor 2 (*TLR2*), and *TLR4* in FHs74 cells (Fig. S6), which could lead to their lack of response to LPS and LTA. Nevertheless, dysregulation of the miR‐223/*NFIA* axis in these intestinal cells has consistently directed to multiple detrimental activities, including increased pro‐inflammatory cytokines, disturbance of homeostasis, induction of apoptosis, and inhibition of the cellular replenishing mechanism.

In summary, our study has provided evidence on the importance of the miR‐223/*NFIA* network in intestinal tissues affected by NEC and its probable contribution to disease pathophysiology. Despite advances in neonatal care over the past decades, morbidity and mortality outcomes of NEC have remained disappointing [2, 42]. Development of novel therapeutic tactics, such as targeting miRNAs using specific antagonists or mimics and genetically engineered extracellular vesicles, has been proposed and under preclinical and clinical trials for various diseases [43, 44]. Our findings provide new insights into the molecular mechanisms of NEC relating to regulatory pathways involving miRNA. The pathways suggested by our results offer new targets for diagnostic and therapeutic options not only for NEC but also for other conditions with underlying intestinal inflammation.

## Materials and methods

### Clinical specimen

Intestinal specimens were collected during surgery from infants with stage III NEC (*n* = 10) in the Prince of Wales Hospital, a tertiary hospital affiliated with the Chinese University of Hong Kong [8]. These samples were histologically examined to confirm NEC diagnosis. In addition, Surg‐CTL tissues were obtained from infants who underwent intestinal surgery because of noninflammatory conditions, including congenital small bowel atresia (*n* = 7), intestinal obstruction (*n* = 2), and elective closure of ileostomy (*n* = 1).

### Ethics statement

Written informed consents were obtained from the parents for all cases. This study has been approved by the Joint Chinese University of Hong Kong and New Territories East Cluster Clinical Research Ethics Committee (2014.159). The study methodologies conformed to the standards set by the Declaration of Helsinki.

### Cell culture

Human epithelial cell lines Caco‐2 (colon carcinoma) and FHs74 (fetal small intestine), and HEK293 (embryonic kidney) cells were obtained from the American Type Culture Collection (ATCC, Manassas, VA, USA). Caco‐2 was cultured in Eagle's Minimum Essential Medium (EMEM; ATCC) supplemented with 20% FBS (Gibco, Carlsbad, CA, USA). FHs74 was cultured in Hybri‐Care Medium containing 10% FBS (Gibco) and 10% epidermal growth factor (EGF; PeproTech, Rocky Hill, NJ, USA). HEK293 was grown in EMEM (ATCC) containing 10% FBS (Gibco).

### Identification of target gene and luciferase reporter assay

Potential target genes of miR‐223 were predicted by six databases: miRTarBase 4.0, DIANA TOOLS TarBase v7.0, DIANA‐microT web server v5.0, TargetScan Human Release 6.2, microRNA.org, and miRDB as previously described [9]. The characteristics of these databases are summarized in Table S4. The reporter plasmid was constructed with the full‐length 3′UTR of *NFIA* (NM_005595.1) cloned into the pEZX‐MT05 plasmid (GeneCopoeia, Rockville, MD, USA). Site‐directed mutation [16] of the binding site was generated using the Q5‐site‐directed mutagenesis kit [New England Biolabs (NEB), Ipswich, MA, USA] and confirmed by DNA sequencing (BigDye Terminator v3.1 Cycle Sequencing Kit; Applied Biosystems, Forster City, CA, USA). All primers used are listed in Table S5. Mimic miR‐223 (mirVanaTM miRNA Mimics; Ambion, Austin, TX, USA) or scrambled mimic control (mimic‐CTL; mirVanaTM miRNA Mimics; Ambion) and reporter plasmids were cotransfected into the Caco‐2 or HEK293 cells using Lipofectamine® 3000 (Invitrogen, Carlsbad, CA, USA). Luciferase activities of reporter proteins Gaussia luciferase (Gluc) and secreted alkaline phosphatase (SEAP) were measured (Secrete‐Pair Dual Luminescence Assay Kit; Genecopoeia). The luciferase activity of Gluc was normalized with SEAP. The transfection of each miRNA mimics was performed in triplicate in each set of experiment.

HEK293 cells or Caco‐2 cells were transfected with *NFIA* 3’UTR reporter plasmid (pEZX‐MT05) using Lipofectamine® 3000 (Invitrogen) in 6‐well plates. After 48 h, the cells were split into a new 6‐well plate in selection medium containing G418 (0.8 mg·mL^−1^; Sigma‐Aldrich, Saint Louis, MO, USA). After 21 days, selected cells stably harboring the exogenous plasmids were expanded in a 75‐cm^2^ flask, and seeded in a 24‐well plate for luciferase reporter assay. Mimic miR‐223, mimic miR‐431 (an irrelevant miRNA), and mimic‐CTL (all mimics from *mir*Vana™ miRNA Mimics; Ambion) were transfected into these cells with Lipofectamine® 3000. After incubation for 48 h, the medium was collected for measurement of luciferase activity.

### Overexpression of miR‐223 upon LPS or LTA treatment

Caco‐2 cells or FHs74 cells were seeded in 24‐well plates and incubated for 16 h before transfection. Mimic miR‐223 or mimic‐CTL was introduced into the cells by Lipofectamine RNAiMAX (Invitrogen). After 24 h, the cells were treated with lipopolysaccharide from *Escherichia coli* 0127:B8 (LPS; Sigma‐Aldrich) or lipoteichoic acid (LTA) from *Staphylococcus aureus* (LTA; InvivoGen, San Diego, CA, USA) at 50 µg·mL^−1^ for 24 h [45, 46, 47].

### Western blot analysis

Caco‐2 cells were lysed with RIPA buffer (Sigma‐Aldrich) supplemented with 1× protease inhibitor (Roche, Basel, Switzerland) and 1X phosphatase inhibitor (Roche) after transfection with mimic miR‐223 or mimic‐CTL. Twenty microgram of proteins was separated by electrophoresis using 10% polyacrylamide gel (Bio‐Rad, Hercules, CA, USA). The proteins were transferred onto nitrocellulose membrane (GE Healthcare, Chicago, IL, USA) and incubated with rabbit anti‐NFIA (1 : 2000; Thermo Fisher, Waltham, MA, USA, Cat. No. PA5‐52252) or rabbit anti‐GAPDH (1 : 5000; Cell Signaling, Danvers, MA, Cat. No. CST5174) in 4 °C overnight. The membranes were then incubated with anti‐rabbit horseradish peroxidase‐conjugated secondary antibody (Cell Signaling, Cat. No. CST7074) for 1 h in room temperature. Chemiluminescent signals were detected by the ECL Prime Western Blotting Detection Reagent (GE Healthcare) and analyzed by the imagej software (National institutes of Health, Bethesda, MD, USA). GAPDH was used as the loading control.

### Real‐time polymerase chain reaction of miRNA and mRNA

miRNA was extracted from intestinal tissues, Caco‐2 cells, or FHs74 cells using the miRNeasy Mini Kit (Qiagen, Hilden, Germany), and reverse‐transcribed using the High‐Capacity cDNA Reverse Transcription Kit (Applied Biosystems) and microRNA RT primers (Applied Biosystems). PCRs were performed using the 7300 Real‐Time PCR System (Applied Biosystems) with TaqMan MicroRNA Assays (Applied Biosystems, hsa‐miR‐223, assay ID: 002295, UGUCAGUUUGUCAAAUACCCCA; U6 snRNA, assay ID: 001973), cDNA templates, and Universal Master Mix (Applied Biosystems). The reaction was performed in duplicate at: one cycle of 95 °C for 10 min, 45 cycles of 95 °C for 15 s, and 60 °C for 1 min. U6 was amplified as an internal control. All reactions were performed in parallel with no‐template control reactions. The level of specific miRNAs was expressed as the relative expression to U6/ *GAPDH*.

Total RNA was extracted from intestinal tissues, Caco‐2, or FHs74 using the RNeasy Mini Kit (Qiagen). cDNA was generated by the High‐Capacity cDNA Reverse Transcription Kit (Applied Biosystems). PCRs were performed using the 7300 Real‐Time PCR System (Applied Biosystems) with TaqMan probes (Applied Biosystems; Table S6), cDNA templates, and TaqMan Gene Expression Master Mix (Applied Biosystems). The reaction was performed in duplicate at: one cycle of 50 °C for 2 min, one cycle of 95 °C for 10 min, 40 cycles of 95 °C for 15 s, and 60 °C for 1 min. *GAPDH* was amplified as the internal control.

### Cell proliferation by MTS assay

Caco‐2 or FHs74 was seeded in 96‐well plates at the density of 2.5 or 4.0 × 10^3^ cells and transfected with mimic miR‐223 or mimic‐CTL. Cell proliferation was measured daily for 5 days using the CellTiter 96® AQueous One Solution Cell Proliferation Assay Kit containing 3‐(4,5‐dimethyl‐2‐yl)‐5‐(3‐carboxymethoxyphenyl)‐2‐(4‐sulfophenyl)‐2H‐tetrazolium (MTS) (Promega, Madison, WI, USA). The absorbance was measured at 490 nm with the PerkinElmer VICTOR^3^ Multilabel Counter Spectrophotometer (PerkinElmer, Waltham, MA, USA).

### Apoptosis assay by flow cytometry

Caco‐2 cells were transfected with mimic miR‐223 or mimic‐CTL for 24 h before treatment with LPS or LTA for 24 h, whereas transfected FHs74 cells were incubated for 48 h and treated with LPS or LTA for 48 h. The percentage of apoptotic and total dead cells were measured by the Apoptosis Detection Kit I (BD Biosciences, Franklin Lakes, NJ, USA) containing phycoerythrin (PE) annexin V and 7‐amino‐actinomycin (7‐AAD) reagents, and analyzed by the FACSCalibur/LSRFortessa Cell Analyzer and CellQuest/FACSDiva software packages (BD Biosciences).

### Network analysis

The MetaCore Analysis Suite (Clarivate Analytics, Philadelphia, PA, USA) was used to map regulatory networks of validated target genes downstream of the miR‐223/*NFIA* axis. Multiple algorithms were applied to show direct gene–gene interaction and the shortest path for interactions.

### Statistical analysis

Expression levels of regulatory genes in NEC and Surg‐CTL tissues were compared using the unpaired *t*‐test (two‐sided). Correlation analyses between miR‐223 or *NFIA* and the regulatory genes were performed using Spearman’s correlation test. Data of luciferase reporter assay, western blot and cell apoptosis assays were analyzed by the paired *t*‐test (two‐sided). The proliferation of mimic miR‐223‐ or mimic‐CTL‐transfected cell lines was compared by two‐way ANOVA with Bonferroni’s correction. *P* values ≤ 0.05 were considered statistically significant. All data are presented as mean ± SEM. Statistical analyses were performed with the graphpad prism 5.0 software (La Jolla, CA, USA).

## Conflict of interest

The authors declare no conflict of interest.

## Author contributions

KYYC, KL, and PCN designed the study; YZW and KTL collated the data and carried out data analyses; HSL, KHL and YHT carried out patient recruitment and collection of clinical data; HSL and KL contributed to the data analysis; YZW, KYYC and KL wrote the manuscript. All authors have read and approved the final submitted manuscript.

## Supporting information


**Table S1**. Clinical characteristics of NEC and Surg‐CTL infants.
**Table S2**. Top candidate target genes of miR‐223.
**Table S3**. A brief summary of functions of potential regulatory genes.
**Table S4**. Bioinformatics tools for miRNA prediction.
**Table S5**. Primers used for site‐directed mutagenesis and DNA sequencing.
**Table S6**. Taqman assays used for target gene validation and miR‐223 functional study.
**Fig. S1**. Specific binding of mimic miR‐223 to 3’UTR of *NFIA* in stably transfected HEK293. HEK293 cells were constructed to stably express wild type 3’UTR *NFIA* (WT) reporter gene and were co‐transfected with (i) mimic‐CTL or (ii) mimic miR‐223, or (iii) the irrelevant mimic miR‐431 (negative control). Luciferase activity was measured after incubation for 48 hours. Statistical analysis was performed by paired‐*t* test. The data were presented as mean ± SEM, *n* = 4.
**Fig. S2**. Correlation between miR‐223 or *NFIA* and *IL6* in NEC tissues. There were no significant correlations between expression levels of *IL‐6* and miR‐223 or *NF1A*.Correlation analysis was performed by Spearman correlation test. r represents Spearman correlation coefficient. *n* = 10 for each group.
**Fig. S3**. Expression of *TNF* in Caco‐2 cells transfected with mimic‐CTL or mimic miR‐223, and stimulated with LPS or LTA. The level of TNF was not affected by overexpression of miR‐223, with or without exposure to LPS or LTA. Gene expression was normalized with *GAPDH*. Statistical analysis was performed by paired‐*t* test. The data were presented as mean ± SEM, *n* = 5.
**Fig. S4**. Expressions of *NFIA* and targeted downstream genes were quantified by qPCR in EC tissues. Results were presented as fold change of respective expressions in NEC *versus* Surg‐CTL. Comparisons of all individual regulatory genes between NEC and Surg‐CTL were statistically significant (*P *< 0.05, *n* = 10, Unpaired‐*t* test).
**Fig. S5**. (A) Representative flow cytometric dot‐plot of mimics‐ transfected Caco‐2 cells upon LPS or LTA stimulation. Apoptosis and total cells death of mimic‐CTL and mimic miR‐223‐transfected Caco‐2 cells upon stimulation with LPS or LTA were measured by flow cytometry. The lower right (LR) quadrant represented apoptotic cells; the upper right quadrant (UR) plus lower right quadrant (LR) were total cell death. (B) Representative flow cytometric dot plot of mimics‐ transfected FHs74 cells upon LPS or LTA stimulation. Apoptosis and total cells death of mimic‐CTL and mimic miR‐223‐transfected FHs74 cells upon stimulation with LPS or LTA were measured by flow cytometry. The lower right (LR) quadrant represented apoptotic cells; the upper right quadrant (UR) plus lower right quadrant (LR) were total cell death.
**Fig. S6**. Expression of *TLR2* and *TLR4* in Caco‐2 cells and FHs74 cells. Expression of *TLR2* and *TLR4* were measured by qPCR. Gene expressions were normalized with *GAPDH*. *n* = 1 for each group.

## Data Availability

The data that support the findings of this study are openly available in Gene Expression Omnibus at https://www.ncbi.nlm.nih.gov/geo/query/acc.cgi?acc=GSE68054, reference number GEO:GSE68054.
