# Supplementary material for: The miR‐223/nuclear factor I‐A axis regulates inflammation and cellular functions in intestinal tissues with necrotizing enterocolitis
Source: FEBS Open Bio. 2021 Jun 1;11(7):1907–20. doi: 10.1002/2211-5463.13164 (PMC8255851; doi:10.1002/2211-5463.13164)
Supplement: Supplementary file 1 — Table S1. Clinical characteristics of NEC and Surg‐CTL infants. Table S2. Top candidate target genes of miR‐223. Table S3. A brief summary of functions of potential regulatory genes. Table S4. Bioinformatics tools for miRNA prediction. Table S5. Primers used for site‐directed mutagenesis and DNA sequencing. Table S6. Taqman assays used for target gene validation and miR‐223 functional study. Fig. S1. Specific binding of mimic miR‐223 to 3’UTR of NFIA in stably transfected HEK293. HEK293 cells were constructed to stably express wild type 3’UTR NFIA (WT) reporter gene and were co‐transfected with (i) mimic‐CTL or (ii) mimic miR‐223, or (iii) the irrelevant mimic miR‐431 (negative control). Luciferase activity was measured after incubation for 48 hours. Statistical analysis was performed by paired‐t test. The data were presented as mean ± SEM, n = 4. Fig. S2. Correlation between miR‐223 or NFIA and IL6 in NEC tissues. There were no significant correlations between expression levels of IL‐6 and miR‐223 or NF1A.Correlation analysis was performed by Spearman correlation test. r represents Spearman correlation coefficient. n = 10 for each group. Fig. S3. Expression of TNF in Caco‐2 cells transfected with mimic‐CTL or mimic miR‐223, and stimulated with LPS or LTA. The level of TNF was not affected by overexpression of miR‐223, with or without exposure to LPS or LTA. Gene expression was normalized with GAPDH. Statistical analysis was performed by paired‐t test. The data were presented as mean ± SEM, n = 5. Fig. S4. Expressions of NFIA and targeted downstream genes were quantified by qPCR in EC tissues. Results were presented as fold change of respective expressions in NEC versus Surg‐CTL. Comparisons of all individual regulatory genes between NEC and Surg‐CTL were statistically significant (P < 0.05, n = 10, Unpaired‐t test). Fig. S5. (A) Representative flow cytometric dot‐plot of mimics‐ transfected Caco‐2 cells upon LPS or LTA stimulation. Apoptosis and total cells death o [file FEB4-11-1907-s001.pdf]

Supplemental Table 1. Clinical Characteristics of NEC and Surg-CTL Infants

Infants	Surg-CTL	NEC	NEC vs Surg-CTL, *P*
**Birthweight, gram**	2113 (1813 -2773)	1080 (821-2090)	0.070
**Gestational age, week**	34.1 (32.3-37.9)	28.9 (26.3-34.7)	0.082
**Gender, male**	5 (50%)	5 (50%)	1.000
**Apgar scores:**			
**1 min**	8 (7-9)	7 (4-8)	0.129
**5 min**	9 (9-9)	8 (7-10)	0.086
**Postnatal age at the onset of illness, day**	3 (1-78)	29 (15-45)	0.129
**Duration of disease onset to surgery, hour**	23 (13-35)	48 (29-64)	0.061
**No. deceased**	1	3	0.582
**Duration of hospitalization, day**	**91 (30-182)**	**205 (134-251)**	***0.014***

n = 10 per group. Results are expressed as number (%) or median (interquartile range). *P* values of Fisher's exact test or Mann-Whitney U test are listed. [1]

Supplemental Table 2. Top candidate target genes of miR-223

Gene Symbol	Fold change in microarray data	miRTar Base	DIANA TOOLS TarBase v 7.0	DIANA-microT web server v5.0	TargetScan Human Release 6.2	microRNA.org	mirDB
NFIA	**0.48**	**Yes**	**Yes**	**Yes**	**Yes**	**Yes**	**Yes**
SCARB1	**0.39**	**Yes**	**Yes**	**Yes**	**Yes**	**Yes**	**No**
ICAM1	**3.71**	**No**	**No**	**Yes**	**No**	**No**	**No**

The binding of miR-223 and 3'UTR of selected potential target genes was predicted by 6 databases: miRTarBase 4.0, DIANA TOOLS TarBase v7.0, DIANA-microT web server v5.0, TargetScanHuman Release 6.2, microRNA.org, and miRDB.

Supplemental table 3. A brief summary of functions of potential regulatory genes

**Gene symbol**	**Gene name**	**Function**	**Disease/cell type**	**Ref.**
GNA11	G Protein Subunit Alpha 11	Regulates contraction of gut smooth muscle through mediating Ca^{2+} mobilization.<br><br>Regulates intestinal homeostasis, proliferation, apoptosis, differentiation and maturation of Paneth cells.	Human smooth muscle cells<br><br>Intestinal epithelia cell-specific Gαq/Gα11 knockout mice	[2, 3]<br><br>[4]
MYLK	Myosin Light Chain Kinase	Modulates smooth muscle contraction through phosphorylating light chain of smooth muscle myosin or directly binding with actin.<br><br>Increases permeability of intestinal epithelial barriers under regulation of Tumor necrosis factor receptor 2 (TNFR2) signaling pathway.<br><br>Increased in intestinal tissues of NEC patients, regulates intestinal permeability *via* tight junction proteins.	Smooth muscle tissues from chicken gizzard<br><br>*Tnfr*$2^{-/-}$ mice<br><br>NEC	[5]<br><br>[6]<br><br>[7, 8]
MYOM1	Myomesin 1	Elastic protein expressed in muscle tissues and plays roles in contractile of muscle.	Human sarcomeres	[9]
NFIA	Nuclear factor I A	Suppresses proliferation of glial precursor by repressing expression of p21, a key regulator of cell cycle and proliferation.<br><br>Promotes differentiation of myeloid progenitors to immunosuppressed Myeloid progenitors-derived suppressor cells (MDSCs). Deficiency of Nfia in myeloid cell attenuates immunosuppressive cytokine IL-10 and improves late-sepsis survival.<br><br>Downregulates protein level of TNF-α, IL-6, and IL-1β in aortic tissue of $AopE^{-/-}$ mice through inhibiting expression of NF-κB.	Glioma<br><br>U87 (human glioma cell line)<br><br>*Nfia*$^{-/-}$ mice<br><br>THP-1 macrophage and $AopE^{-/-}$ mice	[10]<br><br>[11]<br><br>[12]
IL6	Interleukin-6	Stimulates the production of	NEC	[13]

		acute phase proteins in the liver, B cell proliferation and antibody production.<br><br>Stimulates inflammatory response through regulating levels of cell adhesion molecules, such as *Icam1* and *Vcam1*.<br><br>Stimulates production of IL17A and IL22 in colonic lamina propria mononuclear cells of patients with chronic intestinal inflammation.	 <br><br>Mouse model of colitis<br><br><br><br>Chronic intestinal inflammation	 <br><br>[14]<br><br><br><br>[15]
IL8	Interleukin-8	Increases expression in response to LPS and flagellin in immature fetal intestinal cells. Predisposes the premature intestine to inflammation.<br><br>Regulates infiltration of neutrophils in intestinal cells.	NEC<br><br><br><br><br><br><br>Caco-2	[16]<br><br><br><br><br><br><br>[17]
TNF	Tumor Necrosis Factor Alpha	Induces mitochondrial dysfunction and activation of mitochondrial apoptotic responses, leading to intestinal epithelial cell apoptosis during NEC.<br><br>Induces apoptosis of intestinal epithelium cells through binding with tumor necrosis factor receptor 1 (TNFR1) and activating caspase-3.<br><br>Triggers necrosis through activating dynamin-related protein 1 (Drp1).<br><br>Leads to loss of villi and epithelial cells in immature intestine.	NEC and rat intestinal epithelioid-1 cells<br><br><br><br><br>MLCK$^{-/-}$ mice<br><br><br><br><br><br>HT-29 (human colon cancer cell line)<br><br><br>Neonatal mice model of TNF exposure by intraperitoneal injection	[18]<br><br><br><br><br><br>[19, 20]<br><br><br><br><br><br>[21]<br><br><br>[22]
PRKCZ	Protein Kinase C Zeta	Promotes assembly of epithelial tight junctions through regulating phosphorylation of occludin and tight junction protein 1. Reduced expression of	Caco-2 cells and Madin-canine kidney cells	[23]

		PRKCZ results in compromised tight junction integrity.<br><br>Regulates cellular functions such as cell proliferation, TNFα induced apoptosis, adaptive or cell-mediated immune responses *via* Par-4, NF-κB or the Jak1/STAT6 signaling.	 <br>Hela cells, knockout mice model and immune cells	 <br>[24]
RGN (*SMP-30*)	Regucalcin	Protects small intestinal cells from apoptosis when expose to irradiation *via* regulating expression of pro-apoptotic protein Bcl2 associated X protein (BAX) and anti-apoptotic protein B-cell lymphoma 2 (Bcl-2).	SMP-30 knockout mice	[25]

Supplemental Table 4. Bioinformatics tools for miRNA prediction

Database	Experimentally validated	Website	Reference
miRTarBase 4.0	Yes	http://mirtarbase.mbc.nctu.edu.tw/php/index.php	[26]
DIANA TOOLS TarBase v 7.0	Yes	http://www.microrna.gr/tarbase	[27]
DIANA-microT web server v5.0	No	http://www.microrna.gr/webServer	[28, 29]
TargetScanHuman Release 6.2	No	http://www.targetscan.org/vert_61	[30, 31]
microRNA.org	No	http://www.microrna.org	[32-34]
miRDB	No	http://mirdb.org	[35]

Supplemental Table 5. Primers used for site-directed mutagenesis and DNA sequencing

Gene symbol	Orientation	Oligo (Uppercase = target-specific primer)
NFIA	forward	agcccgcacgcGGAATCAATCAAAACAATCGAATTTTG
	reverse	gtcaaaacagacCATCTTCCCCATTTCCATG
sequencing primer (5' to 3')	AAAAGTTTTGAAATGCTGCAC	

Supplemental Table 6. Taqman assays used for target gene validation and miR-223 functional study

Assay identity	Gene symbol	Gene name
Hs01588833_m1	*GNA11*	G protein subunit alpha 11
Hs00364926_m1	*MYLK*	myosin light chain kinase
Hs00187003_m1	*MYOM1*	myomesin 1
Hs00325656_m1	*NFIA*	nuclear factor I A
Hs00951388_m1	*PRKCZ*	protein kinase C zeta
Hs00174131_m1	*IL6*	interleukin 6
Hs00174103_m1	*IL8*	interleukin 8
Hs00174128_m1	*TNF*	tumor necrosis factor alpha
Hs02621280_s1	*TLR2*	toll like receptor 2
Hs00152939_m1	*TLR4*	toll like receptor 4
Hs00275889_m1	*RGN*	regucalcin (SMP30)
Hs99999905_m1	*GAPDH*	glyceraldehyde-3-phosphate dehydrogenase

Supplemental Figure 1. Specific binding of mimic miR-223 to 3'UTR of *NFIA* in stably transfected HEK293

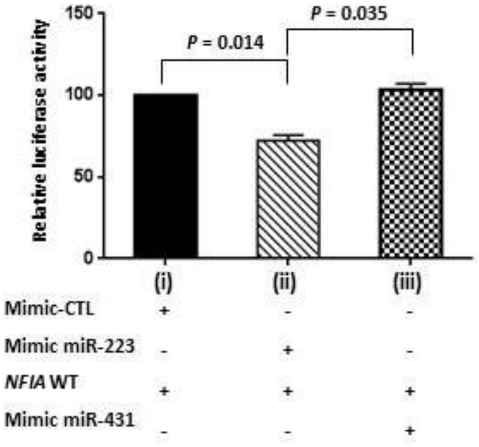

Supplemental Figure 1. Specific binding of mimic miR-223 to 3'UTR of *NFIA* in stably transfected HEK293. HEK293 cells were constructed to stably express wild type 3'UTR *NFIA* (WT) reporter gene and were co-transfected with (i) mimic-CTL or (ii) mimic miR-223, or (iii) the irrelevant mimic miR-431 (negative control). Luciferase activity was measured after incubation for 48 hours. Statistical analysis was performed by paired-*t* test. The data were presented as mean ± SEM, n = 4.

Supplemental Figure 2. Correlation between miR-223 or *NFIA* and *IL6* in NEC tissues.

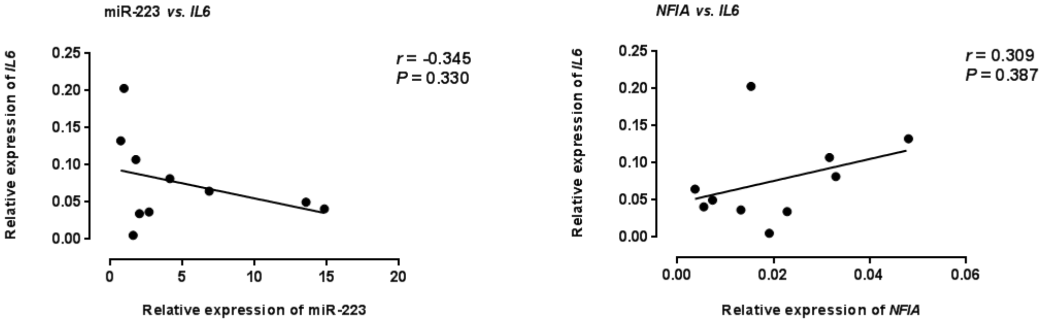

Supplemental Figure 2. Correlation between miR-223 or *NFIA* and *IL6* in NEC tissues. There were no significant correlations between expression levels of *IL-6* and miR-223 or *NF1A*.Correlation analysis was performed by Spearman correlation test. r represents Spearman correlation coefficient. n = 10 for each group.

Supplemental Figure 3. Expression of *TNF* in Caco-2 cells transfected with mimic-CTL or mimic miR-223, and stimulated with LPS or LTA.

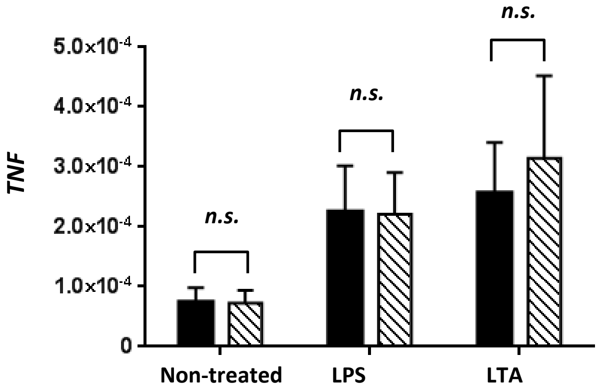

Supplemental Figure 3. Expression of *TNF* in Caco-2 cells transfected with mimic-CTL or mimic miR-223, and stimulated with LPS or LTA. The level of TNF was not affected by overexpression of miR-223, with or without exposure to LPS or LTA. Gene expression was normalized with *GAPDH*. Statistical analysis was performed by paired-*t* test. The data were presented as mean ± SEM, n = 5.

Supplemental Figure 4. Expressions of *NFIA* and targeted downstream genes were quantified by qPCR in NEC tissues.

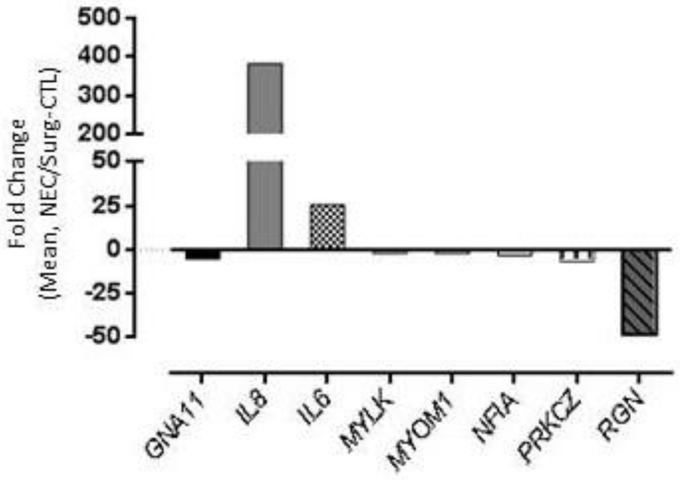

Supplemental Figure 4. Expressions of *NFIA* and targeted downstream genes were quantified by qPCR in NEC tissues. Results were presented as fold change of respective expressions in NEC *versus* Surg-CTL. Comparisons of all individual regulatory genes between NEC and Surg-CTL were statistically significant ($P < 0.05$, n = 10, Unpaired-t test).

Supplemental Figure 5A. Representative flow cytometric dot-plot of mimics-transfected Caco-2 cells upon LPS or LTA stimulation.

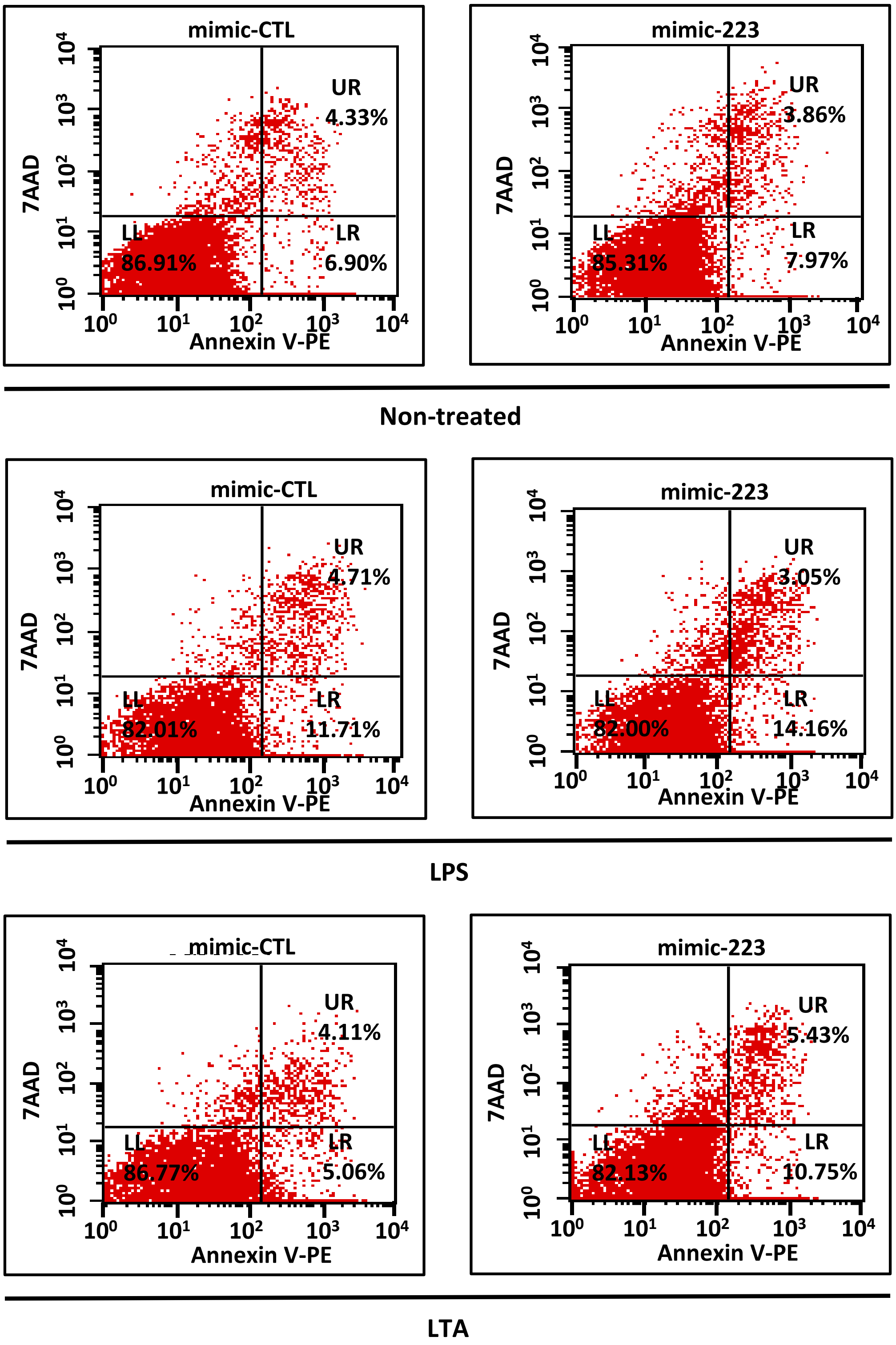

Supplemental Figure 5A. Representative flow cytometric dot-plot of mimics-transfected Caco-2 cells upon LPS or LTA stimulation. Apoptosis and total cells death of mimic-CTL and mimic miR-223-transfected Caco-2 cells upon stimulation with LPS or LTA were measured by flow cytometry. The lower right (LR) quadrant represented apoptotic cells; the upper right quadrant (UR) plus lower right quadrant (LR) were total cell death.

Supplemental Figure 5B. Representative flow cytometric dot plot of mimics-transfected FHs74 cells upon LPS or LTA stimulation.

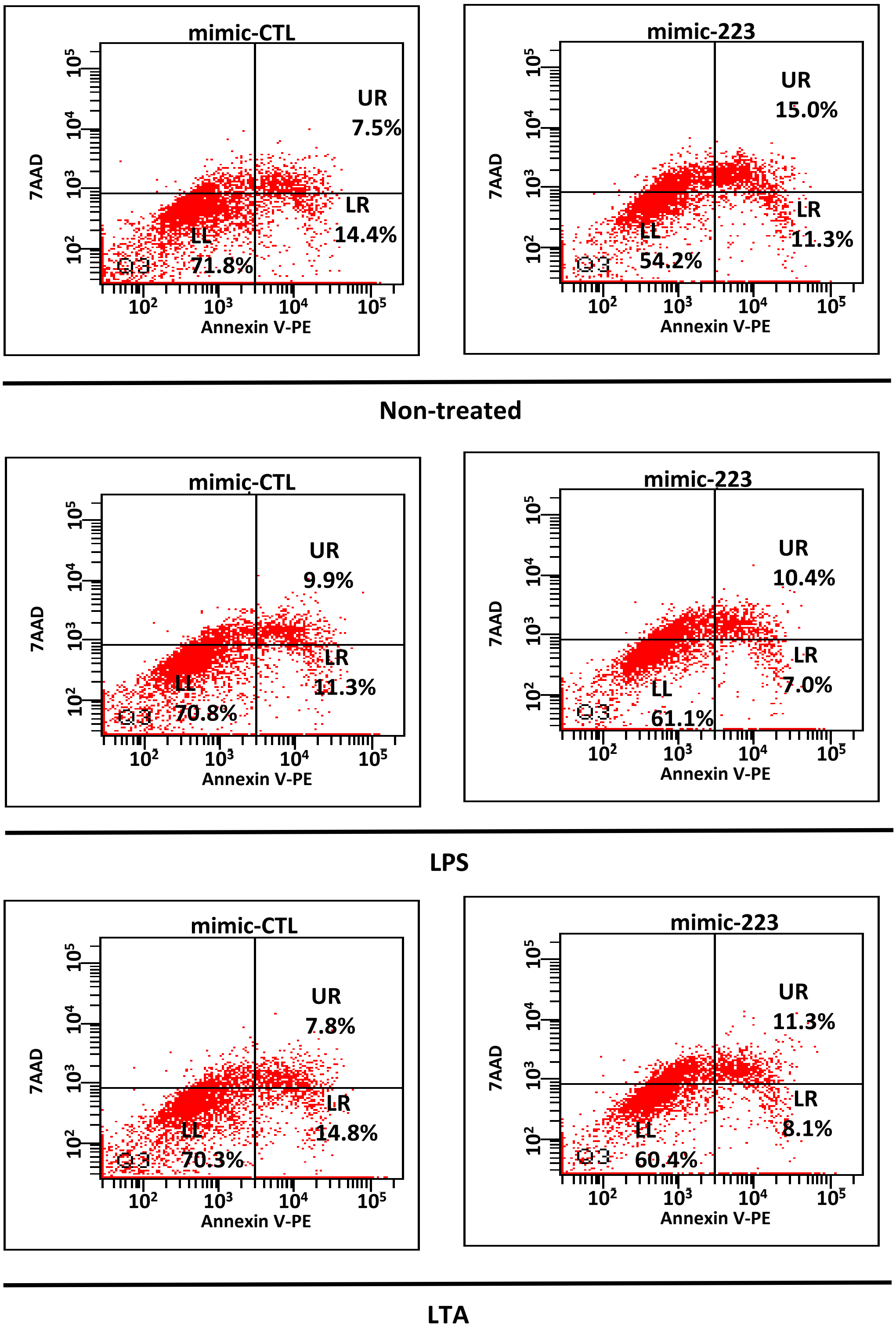

Supplemental Figure 5B. Representative flow cytometric dot-plot of mimics-transfected FHs74 cells upon LPS or LTA stimulation. Apoptosis and total cells death of mimic-CTL and mimic miR-223-transfected FHs74 cells upon stimulation with LPS or LTA were measured by flow cytometry. The lower right (LR) quadrant represented apoptotic cells; the upper right quadrant (UR) plus lower right quadrant (LR) were total cell death.

Supplemental Figure 6. Expression of *TLR2* and *TLR4* in Caco-2 cells and FHs74 cells.

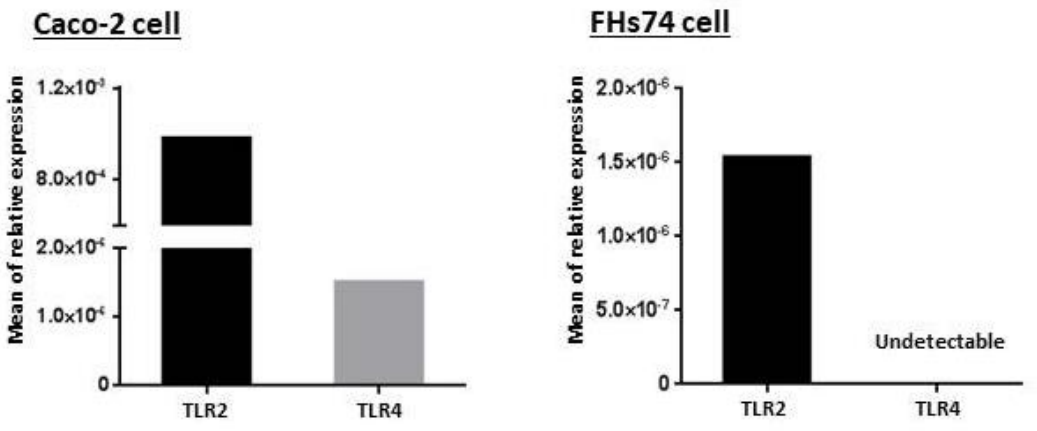

Supplemental Figure 6. Expression of *TLR2* and *TLR4* in Caco-2 cells and FHs74 cells. Expression of *TLR2* and *TLR4* were measured by qPCR. Gene expressions were normalized with *GAPDH*. n = 1 for each group.

References:

[1] Ng, P. C., Chan, K. Y., Leung, K. T., Tam, Y. H., Ma, T. P., Lam, H. S., Cheung, H. M., Lee, K. H., To, K. F. & Li, K. (2015) Comparative MiRNA Expressional Profiles and Molecular Networks in Human Small Bowel Tissues of Necrotizing Enterocolitis and Spontaneous Intestinal Perforation, *PloS one.* **10**, e0135737.

[2] K.Y. Chan, K.T. Leung, Y.H. Tam, H.S. Lam, H.M. Cheung, T.P. Ma, K.H. Lee, K.F. To, K. Li, P.C. Ng, Genome-wide expression profiles of necrotizing enterocolitis versus spontaneous intestinal perforation in human intestinal tissues: dysregulation of functional pathways, Annals of surgery, 260 (2014) 1128-1137.

[3] K.S. Murthy, Signaling for contraction and relaxation in smooth muscle of the gut, Annual review of physiology, 68 (2006) 345-374.

[4] N. Watanabe, H. Mashima, K. Miura, T. Goto, M. Yoshida, A. Goto, H. Ohnishi, Requirement of Galphaq/Galpha11 Signaling in the Preservation of Mouse Intestinal Epithelial Homeostasis, Cellular and molecular gastroenterology and hepatology, 2 (2016) 767-782 e766.

[5] Y. Gao, L.H. Ye, H. Kishi, T. Okagaki, K. Samizo, A. Nakamura, K. Kohama, Myosin light chain kinase as a multifunctional regulatory protein of smooth muscle contraction, Iubmb Life, 51 (2001) 337-344.

[6] L. Su, S.C. Nalle, L. Shen, E.S. Turner, G. Singh, L.A. Breskin, E.A. Khramtsova, G. Khramtsova, P.Y. Tsai, Y.X. Fu, C. Abraham, J.R. Turner, TNFR2 activates MLCK-dependent tight junction dysregulation to cause apoptosis-mediated barrier loss and experimental colitis, Gastroenterology, 145 (2013) 407-415.

[7] S.A. Moore, P. Nighot, C. Reyes, M. Rawat, J. McKee, D. Lemon, J. Hanson, T.Y. Ma, Intestinal barrier dysfunction in human necrotizing enterocolitis, Journal of pediatric surgery, 51 (2016) 1907-1913.

[8] D. Ye, T.Y. Ma, Cellular and molecular mechanisms that mediate basal and tumour necrosis factor-alpha-induced regulation of myosin light chain kinase gene activity, J Cell Mol Med, 12 (2008) 1331-1346.

[9] L. Tskhovrebova, J. Trinick, Making muscle elastic: the structural basis of myomesin stretching, PLoS biology, 10 (2012) e1001264.

[10] S.M. Glasgow, D. Laug, V.S. Brawley, Z. Zhang, A. Corder, Z. Yin, S.T. Wong, X.N. Li, A.E. Foster, N. Ahmed, B. Deneen, The miR-223/nuclear factor I-A axis regulates glial precursor proliferation and tumorigenesis in the CNS, The Journal of neuroscience : the official journal of the Society for Neuroscience, 33 (2013) 13560-13568.

[11] M.B. McPeak, D. Youssef, D.A. Williams, C. Pritchett, Z.Q. Yao, C.E. McCall, M. El Gazzar, Myeloid Cell-Specific Knockout of NFI-A Improves Sepsis Survival, Infection and immunity, 85 (2017).

[12] J.J. Zhao, Y.W. Hu, C. Huang, X. Ma, C.M. Kang, Y. Zhang, F.X. Guo, J.B. Lu, J.C. Xiu, Y.R. Qiu, Y.H. Sha, J.J. Gao, Y.C. Wang, P. Li, B.M. Xu, L. Zheng, Q. Wang, Dihydrocapsaicin suppresses proinflammatory cytokines expression by enhancing nuclear factor IA in a NF-kappaB-dependent manner, Archives of biochemistry and biophysics, 604 (2016) 27-35.

[13] C.J. Hunter, I.G. De Plaen, Inflammatory signaling in NEC: Role of NF-kappaB,

cytokines and other inflammatory mediators, Pathophysiology : the official journal of the International Society for Pathophysiology, 21 (2014) 55-65.
[14] M. Yamamoto, K. Yoshizaki, T. Kishimoto, H. Ito, IL-6 is required for the development of Th1 cell-mediated murine colitis, Journal of immunology, 164 (2000) 4878-4882.
[15] N. Powell, J.W. Lo, P. Biancheri, A. Vossenkamper, E. Pantazi, A.W. Walker, E. Stolarczyk, F. Ammoscato, R. Goldberg, P. Scott, J.B. Canavan, E. Perucha, N. Garrido-Mesa, P.M. Irving, J.D. Sanderson, B. Hayee, J.K. Howard, J. Parkhill, T.T. MacDonald, G.M. Lord, Interleukin 6 Increases Production of Cytokines by Colonic Innate Lymphoid Cells in Mice and Patients With Chronic Intestinal Inflammation, Gastroenterology, 149 (2015) 456-467 e415.
[16] D.O. Son, H. Satsu, Y. Kiso, M. Totsuka, M. Shimizu, Inhibitory effect of carnosine on interleukin-8 production in intestinal epithelial cells through translational regulation, Cytokine, 42 (2008) 265-276.
[17] N. Baregamian, J. Song, C.E. Bailey, J. Papaconstantinou, B.M. Evers, D.H. Chung, Tumor necrosis factor-alpha and apoptosis signal-regulating kinase 1 control reactive oxygen species release, mitochondrial autophagy, and c-Jun N-terminal kinase/p38 phosphorylation during necrotizing enterocolitis, Oxidative medicine and cellular longevity, 2 (2009) 297-306.
[18] C. Gunther, H. Neumann, M.F. Neurath, C. Becker, Apoptosis, necrosis and necroptosis: cell death regulation in the intestinal epithelium, Gut, 62 (2013) 1062-1071.
[19] A.M. Marchiando, L. Shen, W.V. Graham, K.L. Edelblum, C.A. Duckworth, Y.F. Guan, M.H. Montrose, J.R. Turner, A.J.M. Watson, The Epithelial Barrier Is Maintained by In Vivo Tight Junction Expansion During Pathologic Intestinal Epithelial Shedding, Gastroenterology, 140 (2011) 1208-+.
[20] Z.G. Wang, H. Jiang, S. Chen, F.H. Du, X.D. Wang, The Mitochondrial Phosphatase PGAM5 Functions at the Convergence Point of Multiple Necrotic Death Pathways, Cell, 148 (2012) 228-243.
[21] K.S. Brown, H. Gong, M.R. Frey, B. Pope, M. Golden, K. Martin, M. Obey, S.J. McElroy, Tumor necrosis factor induces developmental stage-dependent structural changes in the immature small intestine, Mediators of inflammation, 2014 (2014) 852378.
[22] S. Jain, T. Suzuki, A. Seth, G. Samak, R. Rao, Protein kinase Czeta phosphorylates occludin and promotes assembly of epithelial tight junctions, The Biochemical journal, 437 (2011) 289-299.
[23] J. Moscat, P. Rennert, M.T. Diaz-Meco, PKC zeta at the crossroad of NF-kappa B and Jak1/Stat6 signaling pathways, Cell death and differentiation, 13 (2006) 702-711.
[24] M.J. Goo, J.K. Park, I.H. Hong, A.Y. Kim, E.M. Lee, E.J. Lee, M. Hwang, K.S. Jeong, Increased susceptibility of radiation-induced intestinal apoptosis in SMP30 KO mice, International journal of molecular sciences, 14 (2013) 11084-11095.
[26]S.D. Hsu, Y.T. Tseng, S. Shrestha, Y.L. Lin, A. Khaleel, C.H. Chou, C.F. Chu, H.Y. Huang, C.M. Lin, S.Y. Ho, T.Y. Jian, F.M. Lin, T.H. Chang, S.L. Weng, K.W. Liao, I.E. Liao, C.C. Liu, H.D. Huang, miRTarBase update 2014: an information resource for experimentally validated miRNA-target interactions, Nucleic acids research, 42 (2014)

D78-85.
[27] I.S. Vlachos, M.D. Paraskevopoulou, D. Karagkouni, G. Georgakilas, T. Vergoulis, I. Kanellos, I.L. Anastasopoulos, S. Maniou, K. Karathanou, D. Kalfakakou, A. Fevgas, T. Dalamagas, A.G. Hatzigeorgiou, DIANA-TarBase v7.0: indexing more than half a million experimentally supported miRNA:mRNA interactions, Nucleic acids research, 43 (2015) D153-D159.
[28] M. Reczko, M. Maragkakis, P. Alexiou, I. Grosse, A.G. Hatzigeorgiou, Functional microRNA targets in protein coding sequences, Bioinformatics, 28 (2012) 771-776.
[29] M.D. Paraskevopoulou, G. Georgakilas, N. Kostoulas, I.S. Vlachos, T. Vergoulis, M. Reczko, C. Filippidis, T. Dalamagas, A.G. Hatzigeorgiou, DIANA-microT web server v5.0: service integration into miRNA functional analysis workflows, Nucleic acids research, 41 (2013) W169-W173.
[30] B.P. Lewis, C.B. Burge, D.P. Bartel, Conserved seed pairing, often flanked by adenosines, indicates that thousands of human genes are microRNA targets, Cell, 120 (2005) 15-20.
[31] A. Grimson, K.K. Farh, W.K. Johnston, P. Garrett-Engele, L.P. Lim, D.P. Bartel, MicroRNA targeting specificity in mammals: determinants beyond seed pairing, Molecular cell, 27 (2007) 91-105.
[32] A.J. Enright, B. John, U. Gaul, T. Tuschl, C. Sander, D.S. Marks, MicroRNA targets in Drosophila, Genome biology, 5 (2003) R1.
[33] D. Betel, M. Wilson, A. Gabow, D.S. Marks, C. Sander, The microRNA.org resource: targets and expression, Nucleic acids research, 36 (2008) D149-153.
[34] D. Betel, A. Koppal, P. Agius, C. Sander, C. Leslie, Comprehensive modeling of microRNA targets predicts functional non-conserved and non-canonical sites, Genome biology, 11 (2010) R90.
[35] N. Wong, X. Wang, miRDB: an online resource for microRNA target prediction and functional annotations, Nucleic acids research, 43 (2015) D146-152.
